# An overview of concepts and approaches used in estimating the burden of congenital disorders globally

**DOI:** 10.1007/s12687-017-0335-3

**Published:** 2017-10-11

**Authors:** Sowmiya Moorthie, Hannah Blencowe, Matthew W. Darlison, Joy E. Lawn, Pierpaolo Mastroiacovo, Joan K. Morris, Bernadette Modell, Congenital Disorders Expert Group, A. H. Bittles, H. Blencowe, A. Christianson, S. Cousens, M. W. Darlison, S. Gibbons, H. Hamamy, B. Khoshnood, C. P. Howson, J. E. Lawn, P. Mastroiacovo, B. Modell, S. Moorthie, J. K. Morris, P. A. Mossey, A. J. Neville, M. Petrou, S. Povey, J. Rankin, L. Schuler-Faccini, C. Wren, K. A. Yunis

**Affiliations:** 1 PHG Foundation, 2 Worts Causeway, Cambridge, CB1 8RN UK; 2 Centre for Maternal, Adolescent, Reproductive, and Child Health, London School of Hygiene and Tropical Medicine, London, UK; 3 WHO Collaborating Centre for Community Genetics, Centre for Health Informatics and Multiprofessional Education (CHIME), University College London, London, UK; 4 Coordinating Centre of the International Clearinghouse for Birth Defects Surveillance and Research, Rome, Italy; 5 Centre for Environmental and Preventive Medicine, Wolfson Institute of Preventive Medicine, Barts and the London School of Medicine and Dentistry, Queen Mary University of London, London, UK

**Keywords:** Congenital disorders, Epidemiology, Policy, Public health

## Abstract

Congenital disorders are an important cause of pregnancy loss, premature death and life-long disability. A range of interventions can greatly reduce their burden, but the absence of local epidemiological data on their prevalence and the impact of interventions impede policy and service development in many countries. In an attempt to overcome these deficiencies, we have developed a tool—The Modell Global Database of Congenital Disorders (MGDb) that combines general biological principles and available observational data with demographic data, to generate estimates of the birth prevalence and effects of interventions on mortality and disability due to congenital disorders. MGDb aims to support policy development by generating country, regional and global epidemiological estimates. Here we provide an overview of the concepts and methodological approach used to develop MGDb.

**Electronic supplementary material:**

The online version of this article (10.1007/s12687-017-0335-3) contains supplementary material, which is available to authorized users.

## Introduction

Congenital disorders, often also called birth defects, include “any potential pathological conditions arising before birth, whether they are evident at birth or become manifest later in life” (World Health Organization 1985a; World Health Organization 2000; World Health Organization 2010). Risk factors for congenital disorders include genetic, environmental and wider societal factors. The occurrence and severity of specific congenital disorders are differentially influenced by these risk factors, with some disorders influenced more by genes (e.g. single gene disorders) and others by environmental agents (e.g. those caused by infections). They are an important cause of premature death or life-long disability; however, the absence of local epidemiological data on their birth prevalence and outcomes impedes policy and service development in many countries.

To make up for this deficiency, the Modell Global Database of Congenital Disorders (MGDb) was created to meet the information needs of health policy-makers. It does so through the generation of country, regional and global estimates for selected congenital disorders, by combining general biological principles and available observational data with demographic data. Here we describe the scope of MGDb and the general principles followed in its implementation; more detailed methodology can be found in other articles within this journal supplement and in the UCL repository (Modell et al. 2016).

## Terminology

MGDb uses a set of defined methods to relate demographic data to the known birth prevalence of selected congenital disorders, in order to generate estimates relevant to public health, policy-making and clinical practice. The creation of this tool requires precise definitions of all input data and outputs. Table 1 provides a list of some of the key terms along with their definitions within the context of this tool; a comprehensive glossary can be found in the UCL repository (Modell et al. 2016).

**Table 1:** Terms and definitions

Term	Definition
Miscarriage	Foetal loss before 20 weeks of pregnancy (measured from the last menstrual period)
Foetal death	Death in utero after 20 weeks from the last menstrual period. Used as an indicator of prevalence of stillbirth
Birth	Covers all pregnancy outcomes after 20 weeks of pregnancy (measured from the last menstrual period)
Birth prevalence	Used in place of “incidence” to describe the frequency of new cases as they present to health services. Expressed in terms of affected births per 1000 live births. World Population Prospects (WPP) estimates provide the denominator, and in WPP the term “births” applies only for live births
Baseline birth prevalence	The birth prevalence that would obtain in the absence of any intervention
Actual birth prevalence	Actual births/1000 live births, allowing for the effects of interventions before or during pregnancy
Total birth prevalence	Includes all outcomes of affected pregnancies after 20 weeks’ gestation (termination of pregnancy, foetal death/stillbirth, live birth). Expressed as total affected births /1000 live births
Live birth prevalence	Affected live births/1000 live births
Optimal care	Standard of care available in high-income settings with equitable access to services, at any given point in time
No care	The level of care available in the absence of any supportive medical services
Early mortality	Deaths in children under 5 years of age
Severe disability	Disability plus significantly shortened life expectancy
Less severe disability	Disability with less effect on life expectancy. Ranges from less severe forms of spina bifida to “well on treatment” (e.g. congenital hypothyroidism)
Effective cure	A disorder that has been sufficiently corrected to allow affected individuals to live their lives free from continuing medical care, and to achieve life goals such as independent living, finding a partner, reproductive success, even with some persisting problems. It does not mean complete correction with no residual problems

## Scope of conditions

Only severe, early-onset congenital disorders that cause early death and/or life-long disability in the absence of care and present before 20 years of age are included in MGDb. The disorders currently modelled by MGDb are shown in Table 2. The rationale for modelling these disorders, where data are unavailable, is that their birth prevalence in the absence of access to diagnosis and care (baseline birth prevalence) is relatively constant in any given population. Estimation of the birth prevalence of these conditions can therefore be undertaken assuming that this will vary only with identified factors, such as interventions (e.g. folic acid fortification, prenatal diagnosis, surgical repair). Modelled estimates can be generated on a country-specific annual basis of potential (in the absence of interventions) and actual affected births. Thus, the tool can also be used to estimate the actual effects of any proposed future interventions. Although the same method can be applied to congenital disorders that arise as a result of external risk factors, such as maternal exposure to infection, malnutrition, or teratogens, at present there are insufficient input data at the global level to define a baseline birth prevalence and outcomes of these disorders. This is due to lack of precise estimates of risk associated with these exposures and the fact that risk varies more widely with place and time, requiring country-specific data.

**Table 2:** Groups of congenital disorders modelled by MGDb with principal sources of birth prevalence data

Major category	Intermediate bundle	Diagnostic group	Principal sources for reference baseline birth prevalence rates
Congenital malformations	Neural tube defects	Anencephaly	Elwood et al. (1992), EUROCAT, ICBDSR, literature review and personal communications
		Spina bifida and encephalocele	
	Orofacial clefts	Cleft palate Cleft lip +/− cleft palate	Mossey and Little (2002), Kadir et al. (2016)
	Congenital heart disease^a^ (CHD)	Very severe CHD	EUROCAT
		Severe CHD	
	Other congenital malformations	CNS not neural tube defect	
		Eye	
		Ear, face, neck	
		Respiratory system	
		Digestive system	
		Abdominal wall defects	
		Urinary system	
		Multiple malformations	
		Genital system	
		Limb	
		Congenital hypothyroidism^b^	Modell and Modell (1992)
		Pyloric stenosis	Pedersen et al. (2008), Modell and Modell (1992)
Chromosomal disorders	Down syndrome	Down syndrome	Maternal age calculation
	Other severe autosomal abnormalities	Other trisomies (+13, +18)	
		Other autosomal	Wellesley et al. (2012)
	Sex chromosome disorders	Turner syndrome (XO)	EUROCAT
		Klinefelter syndrome (XXY)	Visootsak and Graham (2006), Morris et al. (2008)
Inherited disorders	Rare single gene disorders	Dominant	Stevenson (1959) Trimble and Doughty (1974), Carter (1977), Baird et al. (1988)
		X-linked	
		Recessive disorders^c^	
	Consanguinity-associated^d^ disorders	Recessive disorders	Bundey and Alam (1993), Bittles and Neel (1994).
	Common autosomal recessive disorders	Sickle cell disorders	Modell and Darlison (2008)
		Thalassaemia	
		Oculocutaneous albinism	Kromberg et al. (1989), Lund and Taylor (2008)
	Genetic risk factors	Rhesus haemolytic disease	Mourant et al. (1976), Bhutani et al. (2013)
		G6PDd kernicterus	World Health Organization (1985b), Howes et al. (2012)

^a^ *CHD* congenital heart defect that usually presents before 20 years of age and would cause premature death or disability in the absence of intervention

^b^Hypothyroidism due to thyroid agenesis or dysgenesis. Hypothyroidism due to iodine deficiency is excluded

^c^Recessive disorders that would occur in the absence of consanguineous marriage and are disadvantageous without any identified compensating selective advantage

^d^Parental consanguinity is associated with an increment in congenital disorders, the increment is mainly due to increased birth prevalence of recessive single gene disorders as parental consanguinity increases the chances that a couple will both carry the same recessive gene variant

The congenital malformation group includes the International Classification of Disease (ICD 10) (World Health Organization 2016) system groups used in most congenital anomaly registries and focuses on isolated malformations (i.e. not those associated with chromosomal disorders or genetic syndromes. Disorders associated with other malformations contribute to the multiple malformations group. Neural tube defects, orofacial clefts and congenital heart defects, are treated separately, as data relating to these malformations are more readily available. The chromosomal disorder group includes all chromosomal disorders that cause substantial disability for the affected person. In MGDb, Edwards and Patau syndrome are treated together as “other trisomies” because their outcomes are very similar. Inherited disorders have been bundled into four broad groups. Rare single gene disorders include dominant, X-linked and recessive disorders that are expected to have a similar collective birth prevalence worldwide, because their gene frequency is mainly determined by the balance between new mutation rate and natural selection, neither of which is thought to vary greatly between populations. The common recessive disorders group includes three conditions (sickle cell disorder, thalassaemia and oculocutaneous albinism) for which there is known global variation in carrier prevalence. Consanguinity-associated disorders refer to the increment of recessive disorders that are associated with parental consanguinity (Corry 2014). Disorders due to genetic risk factors are caused by the interaction of (often very common) DNA variants with other genetic and environmental factors. MGDb includes two early-onset examples (rhesus haemolytic disease of the newborn and neonatal jaundice due to G6PD deficiency) because (a) the underlying mechanisms are exceptionally well understood and (b) they are potentially lethal but can be effectively prevented and/or treated.

## Overview of the methodological approach

**Fig. 1:**
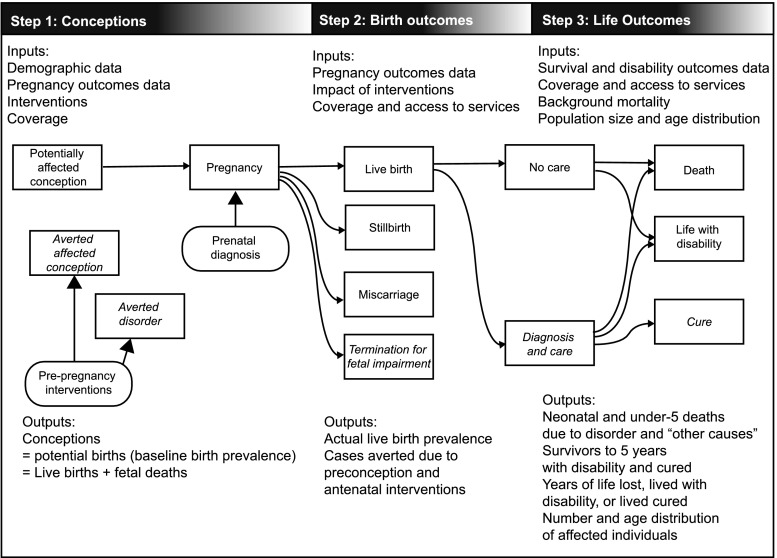
The sequence of events covered in MGDb and stepwise modelling process used to derive estimates for specific congenital disorders. The outcomes that are impacted by interventions are shown in *italics*

The objective of MGDb is to estimate numbers of births affected by one or more congenital disorders, and outcomes in the no-care situation and with current care. Figure 1 shows the range of possible outcomes and their modification by a variety of diagnostic and therapeutic interventions. The effect of some interventions is quantifiable as data relating to their impact are available, for example, folic acid food fortification, the identification of genetic risk and the option of termination of pregnancy. In addition to the impact of particular interventions, their availability and ease of access must be taken into account (Blencowe et al. 2017). Estimates were derived in a stepwise manner, beginning with baseline birth prevalence and outcomes in the absence of interventions (Fig. 1). Once an estimate is available for baseline birth prevalence, outcomes may be calculated based on estimated actual live birth prevalence, estimated access to services and survival with optimal or very limited care.

## Sources of data

### Demographic data

Demographic data not only provide the basis for quantitative epidemiological estimates and the assessment of service needs, they offer an overall picture of many aspects of a population. It is particularly important to recognise the speed of demographic change; all epidemiological estimates need to take account of this dynamic background. The principal source of demographic data is the UN World Population Prospects (UN WPP) (United Nations Population Division 2015), with other data sources used for specific indicators (Bittles and Black 2015; Blencowe et al. 2017; Institute for Health Metrics and Evaluation (IHME) 2015; Inter-agency Group for Child Mortality Estimation (UN-IGME) (2015); UNAIDS 2016). Table 3 provides a summary of the demographic indicators and their sources used to generate estimates for the prevalence and outcomes of congenital disorders.

**Table 3:** Demographic indicators—sources and time period covered by data

Indicator	Use within estimates	Data source	Time-period covered by data
Population (1000 s)	Denominator for prevalence calculations per 100,000 population	UN WPP	1950–2016
Population age distribution	This provides a baseline for estimating present patient numbers	UN WPP	
Annual number of live births (1000 s)	Denominator for all rate calculations (e.g. affected births/1000 births)^a^	UN WPP	
Infant mortality rate (deaths under 1 year per 1000 births)	A basic indicator of health service development used for estimating access to health services and adjusting estimates of mortality and survival for all-cause mortality	UN WPP ^b^	
Under-5 mortality rate (deaths before age 5 per 1000 births	Used for adjusting under-5 deaths due to congenital disorders for all-cause mortality	UN WPP ^b^	
Total fertility rate (TFR) (estimated average births per woman based on current fertility)	Used in estimates for disorders whose prevalence is related to birth number (e.g. rhesus haemolytic disease of the newborn)	UN WPP	
Mean life expectancy (average both sexes)	Provides the basis for calculating years of life affected by congenital disorders (years of life lost, lived with disability or lived cured)	UN WPP	
Maternal age distribution	Proportion of mothers 35 or over is used in calculating potential birth prevalence of maternal-age-related chromosomal disorders	UN WPP	
Stillbirth rate	Used to estimate the contribution of congenital disorders to stillbirths	Blencowe et al. 2017	2000–2014
Population neonatal mortality rate	Total neonatal mortality rates are the denominator for calculating the contribution of congenital disorders to country, regional and global neonatal mortality	UN-IGME	1990–2013
Prevalence of consanguineous marriage (coefficient of consanguinity)	Used for estimating birth prevalence and outcomes of single gene disorders and for adjusting estimated access to services based on infant mortality rate	Bittles and Black (2015)	
Early mortality due to HIV infection	Used for adjusting estimating access to services based on infant mortality rate	Institute for Health Metrics and Evaluation (IHME) (2015)	1990–2013

*UNWPP* United Nations World Population Prospects(United Nations Population Division)

^a^Prevalences of congenital disorders are usually described per 10,000 births in congenital anomaly registries. MGDb uses rates *n*/1000 births because this is the commonest expression in the context of public health

^b^Annual data from 1980, with methodology, is available from http://www.childmortality.org/

### Baseline birth prevalence

The frequency of a disorder in a population is usually described in terms of incidence (number of new cases arising in a defined time-period) and prevalence (number of cases actually present in a given unit of population, e.g. per thousand or per million). However, the incidence of congenital disorders is usually expressed as birth prevalence because although many congenital disorders arise at or around the time of conception, many affected embryos either fail to implant or miscarry in early pregnancy, and so never come to the attention of health services. Therefore, for practical purposes, their prevalence at birth is counted as their incidence, and expressed as rate per 1000 live births. MGDb follows the European Surveillance of Congenital Anomalies and Twins (EUROCAT) convention in counting all pregnancy outcomes after 20 weeks gestation as births. In keeping with ICD-10, all births with any signs of life following separation from the mother, regardless of gestation, are counted as live births.

Baseline, or potential, birth prevalence is the prevalence that would apply in the absence of any intervention. It is the foundation of the MGDb and provides the “envelope” into which all outcomes must fit. It includes stillbirths and live births but excludes miscarriages, and uses “foetal death” (death in utero after 20 weeks’ gestation) as a proxy for stillbirth. Once baseline birth prevalence is known, when appropriate observational data are available baseline births can be allocated to each potential outcome (Fig. 1) thus generating an overall epidemiological picture of the current status of each disorder group.

The baseline birth prevalence for most countries worldwide can be calculated from available data or estimated for many congenital disorders (Table 2). The methodology used to obtain these estimates is described in detail elsewhere (Modell et al. 2016). Briefly, the birth prevalence of maternal age-related chromosomal disorders can be calculated from maternal age distributions for which global data are available (Moorthie et al. 2017). The Hardy-Weinberg (H-W) equation can be used to calculate the birth prevalence of haemoglobin disorders, consanguinity-related disorders, rhesus haemolytic disease and susceptibility to neonatal jaundice due to G6PD deficiency, from available global data on carrier prevalences (Abbas and Yunis 2014; Bittles and Black 2015; Piel et al. 2013a, b). With regard to congenital malformations, there are sufficient observational data available in the literature and from registries on neural tube defects and orofacial clefts to make estimates for countries in every region (Modell et al. 2016; Blencowe et al. 2017), since these severe malformations are evident at birth.

However, data on all other congenital malformations and rare single gene disorders are only available in high-income settings. To generate estimates for congenital malformations for countries outside high-income settings, we have utilised publicly accessible data from the EUROCAT website to obtain average European rates for birth prevalence (Moorthie et al. 2017; Modell et al. 2016). Studies examining ethnic differences have only shown variation for polydactyly, congenital hypothyroidism, orofacial clefts and neural tube defects (Chitty and Winter 1989; Petrini et al. 1997; Terry et al. 1985; Yang et al. 2004). In addition, data from the International Clearinghouse for Birth Defects Surveillance and Research (ICBDSR) indicate little inter-ethnic variation for other malformations. As a result we have assumed that the baseline birth prevalence for these conditions is little affected by ethnicity; therefore, average European rates have been used to generate estimates for countries with no or insufficient observational data (Modell et al. 2016). In the absence of more recent data on the birth prevalence of rare single gene disorders, we have utilised the rates of Baird et al. (Baird et al. 1988) to represent global collective baseline birth prevalences. However, the contribution of specific single gene disorders to collective birth prevalence is known to vary in countries, especially where there are founder effects.

Data on outcomes in the absence of diagnosis and care, and with optimal care (i.e. with the interventions generally available in high-income settings), can be obtained for most groups of congenital disorders. In any setting, a proportion of the population has access to optimal care; however, there is no routinely collected indicator from all countries that provides this information. We have therefore developed a method for estimating the proportion of access to optimal care using infant mortality as a proxy indicator (Modell et al. 2016). Country-specific estimates of outcomes can then be calculated, based on outcomes in the absence of care and with optimal care, and the proportion of the population with access to care (Modell et al. 2016).

There are only three possible outcomes for early-onset congenital disorders in the absence of interventions–foetal death, life with some degree of disability and premature death (Fig. 1). The types and availability of specific interventions have evolved over time, impacting on birth prevalence for specific disorders in different time-periods and the subsequent number of survivors (Modell et al. 2016). Increased access to prenatal diagnosis and termination of pregnancy can act to reduce birth prevalence whilst improvements in diagnosis and care impact on outcomes. Provision of early diagnosis and care can greatly reduce annual numbers surviving with disability for disorders with effective cures. For children with incurable disorders it can both prolong survival and ameliorate the levels of disability. As a result, there may be a steady annual increase in the cumulative number of individuals living with these disorders and requiring appropriate care. The evolution of this effect needs to be quantified in order to assess current and future patient numbers and service needs. A full description of the sources of data on the impact of interventions is available elsewhere (Blencowe et al. 2017; Modell et al. 2016).

### Under-5 mortality

For most congenital disorders mortality is highest within the first 5 years of life, the period for which survival data with no care and with optimal care are most complete and most reliable. To avoid double counting, it is important to allow for the overlaps inherent in multiple causes of death. For assessing total deaths of people with a given disorder, all deaths of affected individuals must be included, whatever the cause. However, when the aim is to assess attributable deaths–deaths that are specifically due to a defined disorder, the background mortality should also be considered. This is because some affected individuals who would have died of their disorder in fact die earlier from unrelated causes, and the proportion of such deaths varies with place and time.

To obtain attributable early deaths, numbers are adjusted for deaths from other causes using country rates for neonatal, infant and under-5 deaths (Modell et al. 2016). The adjustment makes relatively little difference in countries where early background mortality is low, but when background mortality is high a sizeable difference is observed.

### Long-term survival

Table 4 shows the main sources used for estimating survival with limited care, and with optimal care, defined as the best care available around the time the patients were born. The upper age limit of the observational data is also shown. This allows the construction of survival curves in a no-care and an optimal care situation which were then used to estimate number of survivors.

**Table 4:** Main sources of data used for estimating survival with optimal care and no care

Intermediate bundle	Diagnostic group	Optimal survival	Recorded to age	No-care survival
Neural tube defects	Anencephaly	Lethal		Lethal
	Spina bifida and encephalocele	Hunt and Oakeshott (2003) Bowman et al. (2001) Tennant et al. (2010)	30 years 20 years 20 years	Lorber (1971), Laurence and Tew (1971)
Orofacial clefts	Cleft palate Cleft lip +/− cleft palate	Christensen et al. (2004)	Lifetime	(Mossey and Modell 2012)
Congenital heart disease (CHD)	Very severe CHD	Wren and O'Sullivan (2001), Tennant et al. (2010) Wren et al. (2012)	20 years	Macmahon and McKeown (1953), Campbell (1968), Campbell et al. (1957), Baylis and Campbell (1956)
	Severe CHD			
Other congenital malformations	CNS not neural tube defect	Tennant et al. (2010) Skjaerven et al. (1999) Lie et al. (2001)	20 years Lifetime Lifetime	Expert opinion
	Eye			
	Ear, face, neck			
	Respiratory system			
	Digestive system			
	Abdominal wall defects			
	Urinary system			
	Multiple malformations			
	Genital system			
	Limb			
	Congenital hypothyroidism	Assumed normal		Assumed <20 years
	Pyloric stenosis			Lethal
Down syndrome	Down syndrome	Baird and Sadovnick (1988) Baird and Sadovnick (1987) Frid et al. (2004)	Lifetime 1 year	Penrose (1949), Carter and Maley (1958), Stevenson (1959)
Other severe autosomal abnormalities	Other trisomies (+13, +18)	Wu et al. (2013)	5 years	Lethal
	Other autosomal	Estimated 10% < Down		Estimated 10% < Down
Sex chromosome disorders	Turner syndrome (XO)	Price et al. (1986) Stochholm et al. (2006)	60 years	Mortality est. 2× optimal care
	Klinefelter syndrome (XXY)	Bojesen et al. (2004)	Lifetime	Bojesen et al. (2004)
Rare single-gene disorders	Dominant	Costa et al. (1985)	Lifetime	Baird et al. (1988)
	X-linked			
	Recessive disorders			
Consanguinity-associated^d^ disorders	Recessive disorders	Bundey and Alam (1993)	5 yr.	Bittles and Neel (1994)
Common autosomal recessive disorders	Sickle cell disorders	Platt et al. (1994)	Lifetime	Fleming et al. (1979)
	Thalassaemia	Modell et al. (2000), Modell et al. (2008)	45	Modell and Berdoukas (1984)
Genetic risk factors	Rhesus haemolytic disease	Bhutani et al. (2013)	Assumed normal	Stevenson (1959)
	G6PDd kernicterus	World Health Organization (1985b), Bhutani et al. (2013)		World Health Organization (1985b)

Lifetime survival curves with optimal care are only available for some congenital disorders—Down syndrome, spina bifida, orofacial clefts, and haemoglobin disorders. For most other disorders, observational survival data are only available to age 20 or 30 years. Lifetime survival curves were completed for these disorder groups by extrapolating the observed rate of attrition in the last full 5-year interval recorded to 80 years of age. Normal survival is expected for correctable congenital malformations, congenital hypothyroidism and disorders due to genetic risk factors, as optimal care should allow for effective cure or appropriate management of these conditions. Long-term survival in the absence of care is based on literature available from high-income settings in the 1950s for spina bifida, Down syndrome, congenital heart disease and haemoglobin disorders and on expert opinion for most other disorders (Table 4).

Lifetime survival curves are used to project future patient numbers, but they need adjustment for use in calculating current patient numbers, since mortality is usually higher in the early years. The current number of survivors in each age group is largely determined by the services available around their year of birth. Retrospective survival curves were derived from prospective curves, taking into consideration the historical evolution of services, for use in calculating the number and age distribution of patients living at any given time. For this purpose, survival data for each phase in the evolution of care were obtained from the literature (Modell et al. 2016).

### Disability and cure with optimal care and with no care

Definitive cure of conditions is limited to operable congenital malformations. Survivors with most other disorders live with some disability. The survival outcome can range from well (e.g. congenital hypothyroidism with regular replacement treatment) to the very severe (e.g. associated severe physical and mental disability). Due to this diversity, quantification of physical disability is limited to estimates of the proportion of survivors at age 5 with severe disability (including reduced life expectancy), less severe disability (with lesser or no effect on life expectancy) and effectively cured.

Data regarding long-term disability outcomes, depending on the levels of access to care, were obtained through literature review. The proportion of each population without optimal care is assumed to have no access to care. Although we have used the term no-care, this does not refer to the absolute lack of care as this is unlikely to be the case in reality, with supportive care at home or in a facility available in many settings. In addition, we have used estimates for survival with no care based on available data, which in large part is based on survival in the 1950s in “high income” settings (Modell et al. 2016); consequently the survival that we have estimated is likely to be an overestimate for a total absence of care.

## Examples of MGDb outputs by WHO region

The aim of the present exercise is to support health policy makers and practising clinicians at the country level. Outputs are therefore generated to provide a basis for assessing non-financial and financial costs and benefits, and to allow critical comparison with other estimates. MGDb output data are generated for each disorder group by country; these can be aggregated by WHO region. All outputs are expressed in terms of annual numbers and rate per 1000 live births.

### Baseline birth prevalence and actual birth prevalence

**Fig. 2:**
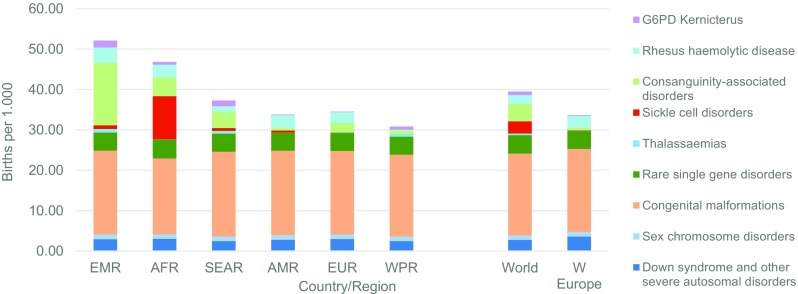
Estimated baseline total birth prevalences of congenital disorder by WHO region. *AFR* African, *AMR* American, *EMR* Eastern Mediterranean, *SEAR* South-East Asian, *WPR* Western Pacific Region, *W Europe* Western Europe

Figure 2 shows the estimated baseline birth prevalence of the groups of congenital disorders by WHO region. All charts also include rates for Western Europe because most currently available interventions are deployed in this sub-region near-equitably at high coverage, and surveillance data are available. Observed outcome data may therefore be used to describe the “power” of each intervention when fully deployed at the population level. There is little inter-regional difference in the baseline prevalence of chromosomal disorders, congenital malformations and baseline single-gene disorders. Most of the inter-region difference is due to genetic disorders where there are inter-country differences in carrier prevalence, or according to the prevalence of consanguinity.

**Fig. 3:**
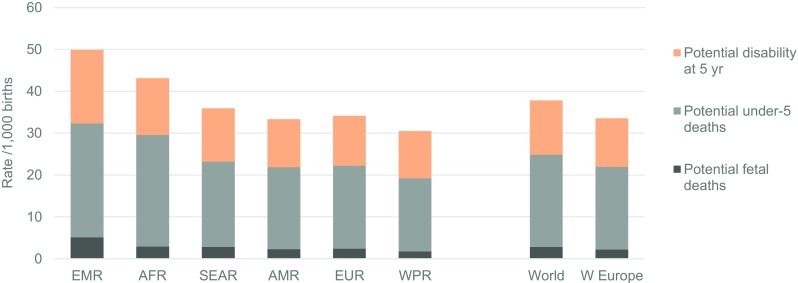
Estimated outcomes of congenital disorders if no intervention, by type of outcome, 2010–2014. Note: The small discrepancy between total affected birth prevalence in the previous chart and total outcomes represents estimated under-5 deaths due to other causes (omitted for the sake of clarity). *AFR* African, *AMR* American, *EMR* Eastern Mediterranean, *SEAR* South-East Asian, *WPR* Western Pacific Region, *W Europe* Western Europe

**Fig. 4:**
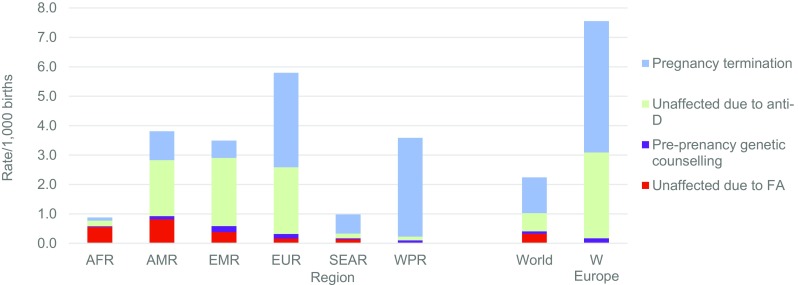
Estimated reduction in affected births per 1000 due to pre-birth interventions, by WHO region, 2010–2014. *AFR* African, *AMR* American, *EMR* Eastern Mediterranean, *SEAR* South-East Asian, *WPR* Western Pacific Region, *W Europe* Western Europe, *FA* Folic acid

Figure 3 shows the corresponding distribution of three possible outcomes in a situation with limited access to care, providing a baseline for assessing the effects of interventions. The estimated effect of pre-birth interventions on affected birth prevalence in 2010–2014 by type of intervention can be seen in Fig. 4. Globally, the largest contributions were from anti-D for rhesus negative mothers and termination of pregnancy for foetal impairment. The effect of pre-pregnancy genetic counselling is relatively small because in most cases risk is only detected retrospectively, i.e. after the diagnosis of an affected child (Modell et al. 2016).

### Birth outcomes, outcome at 5 years of age and effect of interventions

**Fig. 5:**
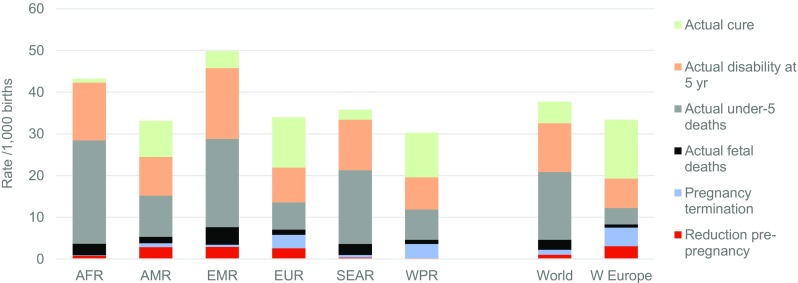
Estimated actual outcomes of congenital disorders by type of outcome, 2010–14. *AFR* African, *AMR* American, *EMR* Eastern Mediterranean, *SEAR* South-East Asian, *WPR* Western Pacific Region, *W Europe* Western Europe

The estimated distribution of actual outcomes in 2010–2014, taking account of interventions and charted within the envelope of potential birth prevalence, can be seen in Fig. 5. By definition, the sum of all outcomes fits into the envelope of baseline birth prevalence. Rates for Western Europe indicate that all available interventions, when deployed at the population level, reduce mortality due to congenital malformations, chromosomal disorders, the two genetic risk factors and haemoglobin disorders by over 80%, but there is far less effect on mortality due to other single-gene disorders. Effective cure is possible for many congenital malformations by paediatric surgery (online resources Fig. 1). Table 5 shows the estimated per cent reduction in unfavourable outcomes due to interventions at 5 years of age, in 2010–2014. The greatest proportional reduction is in under-5 deaths. There is much less reduction in disability—in fact, disability at age 5 due to congenital disorders was increasing in the Eastern Mediterranean, African and South-East Asian regions.

**Table 5:** Per cent reduction in unfavourable outcomes below baseline rates due to interventions, by WHO region, 2010–2014

WHO region	Stillbirths	Under-5 deaths	Disability at 5 years	Total unfavourable outcomes
African	5.2	7.0	−2.1	4.0
American	35.4	49.5	18.8	38.0
Eastern Mediterranean	17.6	22.2	3.4	15.1
European	48.4	67.0	30.1	52.8
South-East Asian	6.8	13.2	5.3	9.9
Western Pacific Region	41.6	58.4	31.5	47.5
World	16.3	26.2	9.9	19.9
Western Europe	66.7	80.0	39.3	65.1

### Mean life expectancy

Table 6 shows disorder-specific mean life expectancy with no care and optimal care, calculated from the full survival curves. The difference between life expectancy with no care and with optimal care measures the survival benefit of care. Mean life expectancy is used to calculate costs of the disorder and the benefits of interventions, in terms of years of life lost, lived with disability or lived cured. The rates in Table 6 represent life expectancy with the disorder in the absence of any other cause of death. These “ideal” rates are adjusted by multiplying local life expectancy divided by a notional optimal life expectancy of 80 years.

**Table 6:** Estimated mean life expectancy with congenital disorders 2010–2014

Major category	Diagnostic group	Mean life expectancy, years		Years gained per affected person
		No care	Optimal care	
Congenital malformations	Anencephaly	0	0	0
	Spina bifida and encephalocele	0.5	41.2	40.7
	Cleft palate Cleft lip +/− cleft palate	4.4	73	68.6
	Very severe CHD			
	Severe CHD	2	6.6	4.6
	CNS not neural tube defect	15.8	63.6	47.8
	Eye	0.5	42.5	42
	Ear, face, neck	39.6	74.7	35.1
	Respiratory system	72.2	72.2	0
	Digestive system	15.6	51	35.4
	Abdominal wall defects	3.8	63.3	59.5
	Urinary system	0.5	65.1	64.6
	Multiple malformations	10.6	69.3	58.7
	Genital system	0.5	37.5	37
	Limb	76.7	76.7	0
	Congenital hypothyroidism	69.8	74.2	4.4
	Pyloric stenosis	11.5	80	68.5
Chromosomal disorders	Down syndrome	0.5	80	79.5
	Other trisomies (+13, +18)	7.7	50.6	42.9
	Other autosomal	0.1	0.1	0
	Turner syndrome (XO)	6.9	45.6	38.7
	Klinefelter syndrome (XXY)	56.8	67.8	11
Inherited disorders	Dominant	66.4	66.4	0
	X-linked	1.3	17.6	16.3
	Recessive disorders	12.1	39.4	27.3
	Consanguinity-associated recessive disorders	6.7	28.1	21.4
	Sickle cell disorders	6.7	28.1	21.4
	Thalassaemia	3	41.5	38.5
	Oculocutaneous albinism	2.4	65.1	62.7
	Rhesus haemolytic disease	30	70	40
	G6PDd kernicterus	1.9	80	78.1

**Fig. 6:**
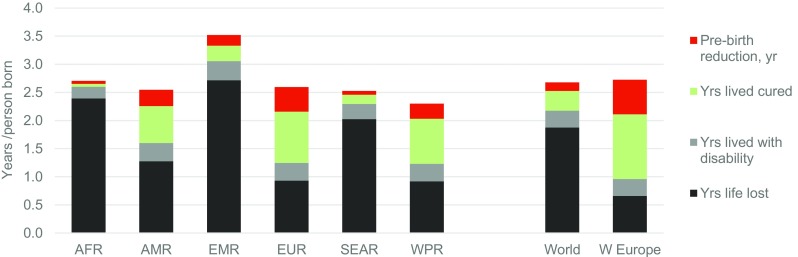
Estimated actual outcomes for total congenital disorders, expressed as years of life lost, lived with disability or lived cured per person in the relevant birth cohort, 2010–2014, by WHO region

Years affected per person born is the same as local mean life expectancy. This provides the envelope for all estimates of outcomes. The world average is 2.5 years affected per person born (range 3.25 for the EMR to 2.2 for WPR). In the absence of intervention, they would be responsible for 2 years loss of life per person born (range 1.7–2.7 years). Figure 6 shows estimated outcomes in terms of years of life lost, lived with disability or lived cured per person born in 2010–2014. The striking reduction in death and disability in Western Europe demonstrates the potential of global implementation of interventions for congenital disorders.

### Estimated number and age distribution of living patients

**Fig. 7:**
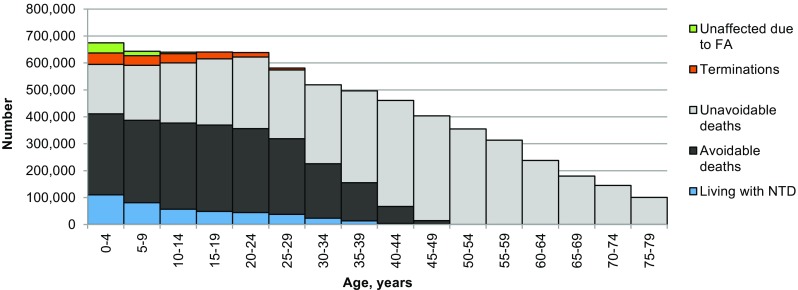
Estimated distribution of outcomes for spina bifida in 2010. The *total outline* shows the number of individuals who would be living with spina bifida if survival equalled the population norm, according to age in 2010 (6.98 million). The *outline* reflects the age distribution of the world population in 2010. The *green fill* shows cases avoided by folic acid food fortification (only 56,000 in 2010 because fortification only started to become policy in the late 1990s). The *orange fill* shows cases avoided by termination of pregnancy (196,000 in 2010). The *grey fill* shows deaths that would have occurred even with best care available when the affected person was born (unavoidable deaths). The great majority of deaths occurred soon after birth, but they appear in all age groups because the chart shows all outcomes. The *black fill* shows the numbers of deaths that occurred because of lack of access to available care (avoidable deaths) (total losses from the current world population due to spina bifida = 6.35 million). The *blue fill* shows estimated numbers living with spina bifida in 2010 (399,000)

To plan for current service needs and project future changes, policy-makers need to know the approximate numbers of persons presently living with specific disorders. This information can only be reliably obtained using a patient register. However, few such registers exist even in high-income settings, even for readily diagnosable disorders such as Down syndrome. The method we have developed extends the principles described above (i.e. consideration of availability of interventions, their impact and evolution along with demographic data) to estimate the number and age distribution of patients currently receiving care. As WPP provides age distribution data from 1950 onwards, it is possible to generate estimates in any year from 1950 onwards, allowing a comprehensive picture of the history of selected disorders up to a chosen year. As an example, Fig. 7 shows global estimates for spina bifida by 5-year age intervals in 2010.

### Future projections

Future projections of the likely effects of implementing interventions, such as folic acid food fortification or prenatal diagnosis with the option of termination of pregnancy are of particular interest to policy-makers. They can be calculated as follows: (a) assuming no change in present policies and (b) assuming worldwide spread of available interventions. Table 7 summarises the estimated long-term effects of different policy decisions for spina bifida at a global level. All of the estimates take account of change in access to care during the time period involved.

**Table 7:** World picture of spina bifida: past and potential future numbers with different future scenarios

Estimate	Past history	Future from 2010		
		No policy change	Global FAF	Global FAF and TOP legal
Potential if no intervention	1950	1970	1990	2010
Unaffected due to FA	2532	3614	5231	6977
Termination of pregnancy	0	0	0	56
Potential with interventions	0	0	28	196
Total deaths	2532	3614	5203	6725
Living with NTD	2486	3490	4989	6347
% of potential unaffected due to FA	47	124	207	399
% of potential avoided by TOP				0.8
% of potential living with NTD			0.5	2.8
Potential if no intervention	1.8	3.4	4.0	5.9

*FAF* folic acid fortification, *TOP* termination of pregnancy

## Discussion

The absence of epidemiological data on congenital disorders has led to the creation of MGDb. However, as with all modelling initiatives, the estimates produced can only be as accurate as the input data, and they are approximations only. We emphasise that our aim is only to produce order of magnitude estimates that can be used as a starting point for service planning. They must not be considered as definitive, and require continuous improvement and refinement as better information becomes available. In undertaking this task, it proved necessary to clarify some concepts and develop some new methods to address gaps in available observational data. Articles within this journal describe some methodological aspects and further details can be accessed via the UCL repository (Modell et al. 2016).

Most estimates generated by MGDb are likely to be underestimates. This is because it is difficult to diagnose many congenital disorders, especially when they cause stillbirth or early death, or in settings with limited diagnostic capacity, which can lead to death and disability being attributed to other causes and a lack of reliable prevalence data. Reliable cause of death data can only be obtained in high-income settings where, by definition, prevention and care are also available. Therefore, if medically certified death rates are assumed to apply globally, early deaths due to congenital disorders will be greatly underestimated. In addition, wherever possible attempts were made to avoid overestimation; some examples are given below. The estimates for single gene disorders are based on older literature and hence only cover conditions diagnosable at that time; but with advances in DNA technology new single gene disorders are being steadily identified (Boycott et al. 2013). The rates for effect of parental consanguinity are largely based on the work of Alan Bittles; but this largely estimates mortality rather than mortality plus disability (Bittles 2003; Bittles and Black 2010), and rates for Rhesus haemolytic disease are lower than those published elsewhere (Bhutani et al. 2013).

### Strengths

The strength of this approach lies in beginning with baseline birth prevalence, which provides the most robust estimates. It provides the envelope for all possible outcomes; consequently, overestimation of one outcome leads to underestimation of another, for example overestimation of mortality leads to underestimation of disability. We have endeavoured to address factors that can lead to overestimation of mortality and prevalence as detailed by Liu et al.(Liu et al. 2012, 2002) and described below.

1. Potential bias towards high prevalence populations, which is a possibility when rates are based on the literature. However, we have endeavoured to use rates based on international registry data. For high-income settings the rates come from EUROCAT, which contains population-based data. Most data for lower-income settings has been obtained from ICBDSR, and most participating registries are hospital-based. This could result in bias due to selective referral of high risk pregnancies, but is unlikely as this often requires routine foetal anomaly scanning. Gene frequency data for haemoglobinopathies, G6PD deficiency and rhesus negativity are based on many large-scale population surveys. Where data were from selected populations, they were excluded.
2. The uncertainty in applying prevalence estimates derived from largely European populations to populations with different fertility patterns, or specific genetic and environmental contexts. For congenital malformations, comparative studies by ethnic group in high-income settings yield comparable rates with two exceptions: (a) a lower birth prevalence of neural tube defects and orofacial clefts among people of Sub-Saharan African origin, and (b) an increased prevalence of congenital malformation syndromes in groups in which consanguineous marriage is customary. Allowances have been made for differences in maternal age distribution, total fertility rate and observed ethnic differences in the birth prevalence of NTD, OFC, CHT and the effect of parental consanguinity by using country-specific data for these conditions and estimating the impact of consanguinity.
3. The fact that some estimates include stillbirths and terminations as well as live births. Due to differences in the definition of stillbirths we have used foetal deaths as an approximation for stillbirths. As many terminations occur before 20 weeks, we have adjusted TOP rates to those that would apply from 20 weeks onwards. In addition, when observed data applied only to live births we added estimates for foetal deaths based on data from registries and the literature.
4. Double counting of infants with more than one congenital disorder. We ensured throughout that numbers and rates apply for affected individuals within the groups specified in Table 1 and created a separate category for cases with more than one congenital malformation.
5. Overestimating mortality due to congenital disorders in lower income settings. Estimates for mortality in a no-care situation are based on historical data from high-income settings and are likely to underestimate a truly no-care situation.

### Weaknesses

As improved data become available from other regions, it will be important to test the assumptions made and to modify them as necessary. The biggest assumptions relate to our estimate of access to care and long-term survival, due to the paucity of data in these areas. A further limitation of this work is a paucity of population-based, cause-specific fatality rate data for varying care settings, necessitating reliance on historical data or expert opinion. There is an urgent need for improved outcome data to verify or amend these estimates. This is an important area for future research, to fully document the long-term impact of living with these conditions and improving the estimates available to more fully quantify the benefits arising from interventions.

### Differences from other estimates

Currently, the only available national and regional estimates for mortality and disability due to congenital disorders were undertaken as part of the Global Burden of Diseases (GBD) study. GBD estimates are limited to congenital anomalies, defined as disorders included in ICD10 chapter VII (the Q chapter)–“Congenital malformations, deformations and chromosomal abnormalities”; that is, they deal only with developmental structural anomalies. Congenital anomaly groups specified in the GBD are Down syndrome, unbalanced chromosomal rearrangements, neural tube defects, orofacial clefts, congenital heart anomalies and other congenital anomalies. These exclude single-gene disorders or genetic risk factors modelled in MGDb; consequently, they were excluded from the comparison.

Table 8 summarises the results of a comparison of estimates of under-5 deaths produced by the GBD (Lopez et al. 2006b) and MGDb. There is reasonable correspondence between estimates for high-income regions and for East and Central Europe where vital registration data are available, but with wide divergences in other regions. The MGDb estimates are consistently higher for most other regions; however, they do fall within the uncertainty range of the GBD data for North Africa and the Middle East and most of sub-Saharan Africa (see online resources Fig. 2).

**Table 8:** Comparison of MGDb and GBD estimates of the contribution of congenital anomalies and congenital disorders to under-5 deaths, by WHO region

WHO region	WPP under-5 deaths/1000		Congenital anomalies: under-5 deaths/1000		% of under-5 deaths attributable to congenital *anomalies*		MGDb estimates (total congenital *disorders*)	
	2005–2009	2010–2014	MGDb 2010–2014	GBD 2012	MGDb % of 2005–2009	GBD % of 2010–2014	Under-5 deaths/1000	% of under-5 deaths 2005–2009
African	114.8	96.4	11.4	4.7	9.9	4.9	27.8	24.2
American	22.9	20.4	6.9	3.2	30.2	15.7	10	43.7
Eastern Mediterranean	68.6	58.3	10.6	5.4	15.4	9.3	21.8	31.8
European	15.9	13.5	3.8	2.5	23.9	18.5	6.6	41.5
South-East Asian	59.1	47.1	11.7	2.7	19.7	5.7	18.6	31.5
Western Pacific Region	21.3	16	4.4	2.8	20.9	17.5	7.4	34.7
World	58.8	49.6	9.0	3.6	15.4	7.3	16.3	27.7
Western Europe	4.7	3.9	2.3	1.14	49.1	29.2	4	85.1

In existing global estimates, confusion about terminology leads to under estimation of the true burden of congenital disorders. For example, estimates of the “congenital” contribution to under-5 mortality published by the GBD study (Lopez et al. 2006a, b) and WHO (Liu et al. 2012) cover only congenital anomalies. They do not include genetically determined disorders (single-gene disorders and disorders due to genetic risk factors), although estimates from MGDb indicate that they contribute to around 50% of under-5 deaths due to congenital disorders. This important point underlines the need for a clearly agreed terminology for community genetics.

It is widely recognised that the average baseline birth prevalence of congenital anomalies is at least 20/1000 while the baseline birth prevalence of congenital disorders (including single-gene disorders and early-onset disorders due to genetic risk factors) is over 37/1000(Baird et al. 1988; Czeizel and Sankaranarayanan 1984; World Health Organization 1985a). Their contribution to early death and disability is masked in lower-income settings by high early mortality from other causes, but it has been estimated that infant mortality can fall below 10/1000 only when interventions for the prevention and care of congenital disorders are in place (Christianson and Modell 2004; World Health Organization 1985a). Consequently, they would be expected to hold a significant place in the Sustainable Development Goals (to end preventable deaths in children) and the WHO strategy for non-communicable diseases (Darmstadt et al. 2016).

## Conclusions

It is possible to overcome the current difficulties in collecting high-quality population-based epidemiological data for congenital disorders in many low- and middle-income countries. The method used here to generate estimates for countries with little or no observational data makes it possible to generate useful order of magnitude estimates by (a) starting from evidence-based estimates of baseline affected birth prevalence; (b) basing estimates for high-income settings on observational data; (c) generating estimates for a baseline no-care situation using the limited observational data supplemented by expert opinion; and (d) using an empirical equation based on infant mortality rates for estimating the proportion of each population with access to the relevant services.

## Electronic supplementary material

ESM 1 (DOCX 24 kb)

## Abbreviations

EUROCAT: European Surveillance of Congenital Anomalies and Twins
ICBDSR: International Clearinghouse for Birth Defects Surveillance and Research
ICD: International Classification of Disease
MGDb: Modell Global Database of Congenital Disorders
NTD: Neural tube defect
OFC: Orofacial cleft
