# Supplementary material for: An overview of concepts and approaches used in estimating the burden of congenital disorders globally
Source: J Community Genet. 2017 Oct 11;9(4):347–62. doi: 10.1007/s12687-017-0335-3 (PMC6167265; doi:10.1007/s12687-017-0335-3)
Supplement: Supplementary file 1 — (DOCX 24 kb) [file 12687_2017_335_MOESM1_ESM.docx]

**Online resources**

Figure 1: Total congenital disorders effectively cured /1,000 births, by cause and WHO region, 2005-09. Over 90% of cure applies for paediatric surgery.

AFR: African, AMR: American, EMR: Eastern Mediterranean, SEAR: South-East Asian, WPR: Western Pacific Region, W.Europe: Western Europe

Figure 2. Comparison of GBD estimates, with range, and MGDb estimates: for under -5 deaths due to congenital anomalies 2010
